# Pharmacological treatment of delayed cerebral ischemia and vasospasm in subarachnoid hemorrhage

**DOI:** 10.1186/2110-5820-1-12

**Published:** 2011-05-24

**Authors:** Diego Castanares-Zapatero, Philippe Hantson

**Affiliations:** 1 Université catholique de Louvain (UCL), Cliniques universitaires Saint Luc, Soins intensifs, Avenue Hippocrate, 10, B-1200 Bruxelles, Belgium

## Abstract

Subarachnoid hemorrhage after the rupture of a cerebral aneurysm is the cause of 6% to 8% of all cerebrovascular accidents involving 10 of 100,000 people each year. Despite effective treatment of the aneurysm, delayed cerebral ischemia (DCI) is observed in 30% of patients, with a peak on the tenth day, resulting in significant infirmity and mortality. Cerebral vasospasm occurs in more than half of all patients and is recognized as the main cause of delayed cerebral ischemia after subarachnoid hemorrhage. Its treatment comprises hemodynamic management and endovascular procedures. To date, the only drug shown to be efficacious on both the incidence of vasospasm and poor outcome is nimodipine. Given its modest effects, new pharmacological treatments are being developed to prevent and treat DCI. We review the different drugs currently being tested.

## Introduction

Delayed cerebral ischemia (DCI) is a common and serious complication following subarachnoid hemorrhage (SAH) after ruptured cerebral aneurismal [1,2]. Although this complication is at times reversible, it may develop into a cerebral infarction [3]. DCI occurs in approximately 20% to 40% [4] of patients and is associated with increased mortality and poor prognosis [5,6]. It is usually caused by a vasospasm [7], which, although preventable, remains a major cause of poor neurological outcome and increased mortality in the course of SAH [4-6].

Vasospasm is defined as a reversible narrowing of the subarachnoid arteries occurring between the third to fifth and fifteenth day after the hemorrhage, with a peak at the tenth day. It is observed in 70% of patients on angiographic scans and causes symptoms in 50% [7-10]. Angiographic vasospasm is defined as evidence of arterial narrowing compared with the parent vessels [11]. It preferentially involves the vessels of the cranial base but also may affect small-caliber vessels or diffusely the entire cerebral vascularization. The severity of vasospasm is variable. The subsequent decrease in cerebral blood flow (CBF) in the spastic arteries leads to DCI, which may develop into cerebral infarction [7,12,13].

The etiology of vasospasm is complex and still poorly understood. Several factors have been shown to be involved, such as endothelial dysfunction, loss of autoregulation, and a hypovolemic component leading to a decrease in CBF [14-16]. At the acute phase, the presence of oxyhemoglobin in the subarachnoid spaces causes a local and systemic inflammatory reaction [17] with activation of platelets and coagulation [8-10]. The products derived from red blood cells (bilirubin) and endothelium (endothelin-1, free radicals) are considered to be mediators of the vasospasm [18-22] Structural anomalies in endothelial and smooth muscle cells also have been reported [23].

Treatments of DCI consist of preventing or minimizing secondary injuries by means of hemodynamic managements, pharmacological agents, and endovascular procedures [12,24,25]. Although these measures result in a decrease in the incidence of vasospasm, the prognostic of DCI remain unchanged [5,24].

Because SAH is frequently accompanied by cerebral autoregulation impairment, hypotension should be avoided. To achieve an adequate cerebral perfusion pressure, triple H therapy was designed to induce volume expansion, rheology improvement, and blood pressure increase. Hence, systolic arterial pressure is increased to approximatively 150-175 mmHg once aneurysm is secured [26]. Before treating aneurysm, it is nevertheless mandatory to maintain systolic blood pressure at lower levels than 150 mmHg. However, there is now evidence suggesting that blood pressure increase is the most important part of those measures because hypervolemia does not have any benefit on cerebral blood flow and tissue oxygenation.

Although triple H therapy reverses deficits associated with vasospasm, it has not been shown to decrease DCI occurrence or mortality [27].

Besides hemodynamic treatment, various pharmacological treatments have been tested [28,29]. Nimodipine is the currently recommended drug [30]. Given its relatively modest effects, new treatments have been developed.

We review recent literature pertaining to the different drugs being used or under evaluation.

## Calcium channel blockers

Nimodipine is a voltage-gated calcium channel antagonist that inhibits calcium entry into smooth muscle cells and neurons. Its lipophilic properties allow it to cross the hematoencephalic barrier. Prophylactic administration of nimodipine was shown to be efficacious in decreasing the risk of secondary ischemia and poor outcome [31,32]. The latest guidelines of the *American Stroke Association *recommend the oral administration of nimodipine at the dose of 60 mg every 4 hours for 21 days starting from the admission into the intensive care unit (Class I, Level of evidence A) [29].

The proof of its efficacy is based on four randomized, placebo-controlled trials of 853 patients, showing an improvement in functional outcome [32-36]. None of the studies were able to demonstrate a reduction in angiographic vasospasm [31]. Its benefits seem to derive from neuroprotective properties rather than its vasodilatory effects. The exact mechanism preventing and limiting the extension of ischemic lesions remains unknown. In experimental models, nimodipine has been shown to attenuate the neuronal calcium increase after cellular ischemia and causing cell death [37].

Whereas calcium is recognized to play a significant role in the occurrence of vasospasm, other elements, such as inflammatory mediators, blood rheology, or microcirculation disturbances, are to be considered. Oxyhemoglobin, for example, causes a decreased activity of potassium channels, which may lead to membrane depolarization and consecutive vasoconstriction [2].

Nimodipine has been shown to be safe [38] and cost-effective [39] without any effect on mortality. Hypotension is a rarely reported side-effect. The current recommendations are based on data pertaining to oral administration of nimodipine. A recent study attempted to show that nimodipine's intravenous use would be associated with similar beneficial effects [40], although this mode of administration is more often linked to hypotension [41].

Among the other tested calcium channel antagonists, nicardipine was shown to decrease symptomatic vasospasm [39,42] but without having any effect on DCI and outcome. The prophylactic use of diltiazem was investigated in a single monocenter study [43]. The rate of favorable outcome was 74.8%.

During endovascular procedures, intra-arterial infusion of nicardipine [44], nimodipine [45], and diltiazem [46] were shown to reduce vasospasm with favorable effects on DCI. However, randomized control studies are still needed (Class IIb, Level of evidence B).

Lastly, two studies showed that prolonged-release nicardipine-loaded polymers implanted upon aneurysmal clipping decreased vasospasm and DCI and improved outcome [38,47]. This mode of administration is promising, although further investigations are necessary.

## Tirilazad

Tirilazad mesylate is a neuroprotective corticosteroid whose efficacy was demonstrated in animal stroke models [48]. It has antioxidant properties that block free radical-induced peroxidation of membrane lipids, which has been shown to facilitate vasospasm. The compound was evaluated in combination with nimodipine in five randomized, double-blind, placebo-controlled trials on a total of 3,821 patients, but no benefit was noted on DCI or outcome [49-52]. Therefore, this drug is not recommended.

## Statins

Statins are inhibitors of 3-hydroxy-3-methylglutaryl coenzyme A, responsible for cholesterol synthesis [53]. In addition, statins display pleiotropic effects, such as anti-inflammatory effects, a stabilizing effect on atheromatous plaques, and anti-adhesive effects on endothelium. Neuroprotective activity also was reported [54,55].

Animal experiments showed a lower incidence of vasospasm when simvastatin therapy was initiated at the time of SAH [56,57]. This beneficial effect is assumed to be due to increased nitric oxide (NO) production on account of NO synthesis induction [58], with subsequent vasodilatation likely to improve CBF.

Several retrospective studies demonstrated that patients treated with statins before SAH presented less DCI and fewer cerebral infarctions [59]. Conversely, other retrospective investigations did not reveal any statin-induced benefits on vasospasm and outcome [60].

Two prospective phase II studies with 80 and 39 patients treated by simvastatin and pravastatin, respectively, revealed a reduction in vasospasm, a decrease in DCI, and an improvement in functional outcome [61,62]. The use of statins deemed safe in both studies.

Nevertheless, the subsequent studies were not able to confirm the benefits of statin therapy. Two prospective, placebo-controlled trials using simvastatin on a small number of patients in addition [63,64] to three observational studies with a historic control revealed no improvement in vasospasm incidence, DCI, or outcome [59,60,65].

Even if the most recent meta-analysis did not confirm the significant effect of statins [66], it should be noted that the trial results are difficult to interpret given the variable disease severity in the control groups and the differing methodologies used. Moreover, it is hazardous to draw conclusions on the basis of four placebo-controlled trials that included only 190 patients. The potential advantage of statins cannot be ruled out. In light of their potential benefits, the current recommendations [29] state that statins may be initiated in patients with SAH (Class IIb, Level of evidence B).

Presently ongoing is a multicenter phase III study on 1,600 SAH patients: STASH (Simvastatin in Aneurysmal Subarachnoid Hemorrhage). This investigation was designed to assess the effects of simvastatin given at a 40-mg dose for 21 days versus placebo. The primary evaluation criterion is functional outcome at 6 months using the modified Rankin disability score (mRS).

## Magnesium sulfate

Magnesium exerts vasodilatory effects by blocking voltage-gated calcium channels. Hypomagnesemia occurs in 38% of SAH patients and is a predictor of DCI [67].

Based on animal experimental models supporting its neuroprotective activity [68], magnesium could be instrumental in improving vasospasm and limiting cerebral ischemia in humans. Although clinical studies have demonstrated that magnesium is safe, they were not able to confirm its efficacy clearly. The first clinical trials showed a trend toward reduced DCI and improved outcome [69]. The randomized, double-blind, placebo-controlled MASH (Magnesium in Aneurysmal Subarachnoid Hemorrhage) study, including 283 patients revealed reduced DCI and improved outcome at 3 months, but the differences with placebo did not reach statistical significance [70]. Another study using the same design and involving 60 patients showed a significant reduction in vasospasm duration assessed by ultrasounds, but no difference in outcome at 6 months [71].

Given that few studies obtained sufficient statistical strength, further clinical trials continue to be undertaken. The multicentre IMASH (Intravenous Magnesium Sulphate for Aneurysmal Subarachnoid Haemorrhage) study reevaluated the effect of magnesium on 327 patients using a prospective, double-blind, placebo-controlled design. No difference in outcome was observed at 6 months, nor was there any effect on clinical vasospasm [72]. The results from another large multicenter study (MASH-II) are expected soon. Even though a few studies reported the occurrence of hypotension, data concerning its impact on DCI and outcome is still lacking [73].

Although magnesium cannot be explicitly recommended at present, it may exert neuroprotective activity, independently from the occurrence of vasospasm. In fact, the concept of predicting DCI in relation to the occurrence of vasospasm must be put into perspective. Given that endothelial dysfunction is the cause of cerebral perfusion problems, magnesium may play an important role, independently from its vasodilatory effects, although its mechanism is not yet understood. It has been shown to be protective in other types of brain injury, such as acute ischemic stroke [74].

## Endothelin-1 antagonist

Endothelin (ET) is a powerful vasoconstrictor [75]. Its receptors are situated on smooth muscle cells (ET receptor ET_A _and ET_B2_) and endothelium (ET receptor ET_B1_). The isoform 1 (ET-1) displays a more significant effect on cerebral arteries, and elevated ET-1 levels were observed in plasma and CSF (cerebrospinal fluid) after SAH [76]. ET-1 has been suggested to largely contribute to vasoconstriction-vasodilatation imbalance during SAH [77,78].

ET-1 receptor blockers have been developed and successfully tested in animals [79]. The first nonselective antagonist (TAK-044) was evaluated, showing a decrease in ischemic events at 3 months in 420 patients [80]. A selective ET_A _antagonist (clazosentan) was shown to decrease the frequency and severity of vasospasm in a preliminary phase IIa study [81]. Three doses of clazosentan were recently tested on 413 patients in a randomized, double-blind, placebo-controlled study (CONSCIOUS-1: Clazosentan to Overcome Neurological Ischemia and Infarction Occurring after Subarachnoid Haemorrhage) [82]. The treatment was initiated within the first 56 hours and continued for 14 days. The aneurysmal treatment was conducted before or in the first 12 hours after administering clazosentan.

A dose-dependent decrease in angiographic vasospasm was observed. No benefit was noted on outcome, although this was not the primary evaluation criterion of the study. In post-hoc analyses, a trend toward improved clinical outcome was reported. Clazosentan was associated with an increased frequency of side-effects, such as hypotension, anaemia, and pulmonary infections. In addition, an increase in mortality was found in the active-treatment group. The majority of deaths were due to peroperative complications. Two phase III studies (CONSCIOUS-2 and CONSCIOUS-3) are currently ongoing in patients treated using clipping or coiling [83].

## Fasudil

Fasudil is a rho-kinase inhibitor, an enzyme involved in the contraction of smooth muscle cells [84]. The inhibition of the rho-kinase pathway causes cellular relaxation. Fasudil, initially investigated in Japan on 276 patients, was shown to reduce vasospasm but without any effect on outcome [85]. However, when administered intra-arterially in combination with the drainage of intracisternal clots and intracisternal urokinase injection, fasudil appeared to reduce the incidence of vasospasm and improve outcome. A recent review of 90 cases seemed to indicate that this procedure was safe and effective on vasospasm and DCI [86]. Further investigations are therefore necessary.

## Antiplatelet therapy

Due to the formation of microthrombi and secretion of thromboxane A2, platelet aggregation may play a role in DCI. Seven randomized and controlled trials involving 1,385 patients tested the effect of antiplatelet agents (acetylsalicylic acid or ticlopidine). However, none revealed any benefit on DCI or patient outcomes [87].

## Enoxaparin

A single study tested the effect of low-molecular-weight heparin following SAH. In a randomized, double-blind, single-center trial of 170 patients, enoxaparin was administered 24 hours after aneurysm treatment and continued for 10 days. There was no benefit on outcome at 3 months [88]. In addition, cerebral bleeding rate was increased with enoxaparin.

## Albumin

The neuroprotective activity of albumin was suggested in different types of brain injury, such as cranial trauma, cerebral ischemia, and SAH [89]. Albumin demonstrated improved CBF in a dog model of SAH [90]. Human data suggest that albumin has protective effects in ischemic stroke [91].

A retrospective study comparing albumin 25% and 0.9% NaCl administered for intravascular filling revealed improved outcome at 3 months in the albumin-treated group, whereas the incidence of vasospasm did not differ [92]. A prospective, multicenter study, Albumin in Subarachnoid Haemorrhage (ALISAH), designed to demonstrate the tolerability and safety of four doses of albumin is currently in progress [93]. This study has been designed to determine the maximally tolerated dose without provoking cardiac decompensation and pulmonary edema. An evaluation of neurological deteriorations is performed at 15 days and 3 months. The toxin-scavenging action of albumin has already been described in numerous diseases [94]. It is possible that albumin acts by scavenging mediators of endothelial dysfunction, such as free radicals.

## Nitric oxide donors

An alteration in NO production is an important mechanism in vasospasm etiology [95,96]. A decrease in NO synthesis during SAH has been noted and is responsible for deficient vessel relaxation and a subsequent decline in CBF [97]. The concept of NO donors was proposed as treatment for refractory vasospasm. Different modes of administration were tested: intravenous, intra-arterial, and intrathecal [2,98]. Intraventricular administration of sodium nitroprusside was shown to improve vasospasm and CBF, although side-effects were common [99]. One study suggested an improvement in outcome [100], whereas another involving a small number of patients did not reveal any effect of transdermal nitroglycerin [101]. Currently, NO donors have a limited place in DCI treatment, and further investigations are needed.

## Erythropoietin

Erythropoietin (EPO) is an amino acid sialoglycoprotein secreted by the kidney and known to play a role in hematopoiesis [102]. EPO receptors have been found in a large number of tissues other than bone marrow, and its neuroprotective role has been suggested [103]. *In vitro *experiments and animal studies showed that EPO enhances neuronal survival under stress situations, such as excitotoxicity [104] and ischemia [105,106]. EPO doses must be sufficiently high due to its weak capacity to cross the blood-brain barrier [107]. The proposed mechanisms are diverse, including anti-inflammatory and anti-apoptotic roles, and modulating NO production [108].

Two double-blind placebo-controlled trials were conducted involving 73 and 80 patients, respectively [109,110]. Tseng et al. showed that patients treated with EPO had a lower incidence of severe vasospasm (27.5 vs. 7.5%), reduced DCI (40 vs. 7.5%), and improved outcome [105]. Even if the number of investigated patients is still low, EPO is considered to be a promising molecule given its beneficial effects at the acute SAH phase and its protective effects at the ischemic phase.

## Intracisternal thrombolytics

An etiological role was attributed to spasmogenic substances released from clots in the subarachnoid spaces. Of note is that the quantity of blood is considered to be a predictor of vasospasm [111]. The intraventricular injection of thrombolytic agents was proposed as treatment. To date, two types of thrombolytics have been tested: urokinase and t-PA (tissue plasminogen activator). The analysis of reported cases suggests a beneficial effect, although it is limited due to the small number of randomized studies [112]. Only one double-blind, placebo-controlled trial assessed the peroperative administration of t-PA on 100 patients [113]. No clear benefit was found with respect to vasospasm and DCI; only patients showing large clots experienced a decrease in vasospasm. The use of this technique requires further prospective studies to define optimal timing, mode of administration, and the type of patients likely to benefit the most. The low incidence of reported complications encourages the undertaking of new studies.

## Conclusions

The poor prognosis of patients with DCI following SAH remains a major issue responsible for death and infirmity. Although our understanding of the physiopathology of DCI and vasospasm has improved, patient outcome has not been significantly modified. Management currently focuses on CBF improvement along with hemodynamic manipulation and endovascular procedures. The only recommended pharmacological treatment is nimodipine.

Although the different compounds tested mostly show a decline in the incidence of radiographic vasospasm, they do not impact on outcome. New pharmacological treatments with neuroprotective effects, such as statins, magnesium, and endothelin inhibitors, revealed promising results. However, the lack of randomized designs and insufficient statistical power of these studies do not allow us to recommend these medications in SAH management at the present time.

The disassociation of vasospasm and clinical outcome also is linked to the fact that DCI occurring after SAH is a multifactorial process without being restricted to arterial narrowing. Effectively, DCI may not only be predicted by cerebral vessels caliber alone; it also may occur in the absence of major vasospasm.

Future investigations should allow us to better understand the mechanisms of endothelial dysfunction, such as oxidative stress, inhibition of vasodilation, and the secretion of vasoconstrictors. The physiopathology of microcirculation dysfunction is all the more complex as unspecific phenomena, such as inflammation, platelet activation, and microthrombi formation. In addition, vasoconstrictors, such as norepinephrine, may have paradoxical effects, and their impact on cerebral microcirculation has not been determined yet.

There is evidence supporting the use of neuroprotective agents. The results of ongoing randomized studies will confirm or not the efficacy of these new treatments. While awaiting potential benefits from neuroprotective treatments, the standard management of intensive care patients using specifically metabolic and ionic control as well as temperature maintenance is still required to preserve the damaged brain. Fever is a common complication and is related to prognosis during the first 2 weeks after SAH [114]. If hyperthermia should be avoided in a patient with increased intracranial pressure, early fever control after SAH could be associated with improved outcome. Recent retrospective data have shown that temperature maintenance above 37°C during the first 2 weeks may be associated with a better outcome [115].

## Competing interests

The authors declare that they have no competing interests.

## Authors' contributions

DCZ and PH wrote the present manuscript and approved its final version.
